# Characterization of a Lithium Disilicate CAD/CAM Material with Firing Temperature-Controlled Translucency

**DOI:** 10.3390/ma18071591

**Published:** 2025-04-01

**Authors:** Alvaro Munoz, Chris Louca, Alessandro Vichi

**Affiliations:** School of Dental, Health and Care Professions, University of Portsmouth, Portsmouth PO1 2QG, UK; alvaro.schiemann@port.ac.uk (A.M.); chris.louca@port.ac.uk (C.L.)

**Keywords:** ceramic, metal-free, CAD/CAM, flexural strength, translucency, lithium disilicate

## Abstract

Lithium disilicates are widely used in restorative dentistry due to their aesthetics, strength, and durability. Increased strength can be achieved by increasing crystal fraction, but this modification also reduces translucency. Recently developed lithium disilicates like Amber Mill claim to offer customizable translucency via firing protocols without changes in flexural strength. This study evaluated whether Amber Mill’s firing protocols produce significant differences in translucency without changing flexural strength. Forty specimens (n = 10) were assessed for translucency using Contrast Ratio (CR) and Translucency Parameter (TP) tests under four firing protocols designed to obtain high translucency (HT), medium translucency (MT), low translucency (LT), and medium opacity (MO). Using the three-point bending test, sixty specimens (n = 15) were tested for flexural strength with the same four firing protocols. The Weibull modulus and characteristic strength were also calculated, and SEM observation was performed. The CR and TP tests revealed statistically significant translucency differences only between MO and LT/MT/HT. Flexural strength ranked as MO > LT > MT > HT, with significant differences observed between MO vs. MT/HT and LT vs. HT. The findings indicate that the recommended firing protocols for the same shaded blocks resulted in limited differences in translucency. Additionally, higher translucencies were associated with reduced flexural strength, highlighting a trade-off between aesthetic and mechanical properties for Amber Mill.

## 1. Introduction

Lithium silicate glass ceramics have become central to restorative dentistry due to their outstanding aesthetic and mechanical qualities. Since the introduction of IPS Empress 2 in the late 1990s, which incorporated 65 vol% lithium disilicate into a glass matrix, significant advancements have been made, particularly with the development of IPS e.max Press. This material improved mechanical properties by incorporating densely packed, elongated lithium disilicate crystals, thereby enhancing crack resistance and toughness [1,2]. Due to their durability, aesthetics, and biocompatibility, these materials have become one of the preferred choices for monolithic restorations, such as crowns, veneers, and inlays [3].

The advent of Computer-Aided Design/Computer-Aided Manufacturing (CAD/CAM) technology has further revolutionized indirect restorations by allowing for greater precision and efficiency [4,5]. IPS e.max CAD, launched in 2006, introduced a pre-crystallized block that could achieve desirable translucency and strength after heat treatment, thereby expanding the aesthetic and functional possibilities in dental restorations [6,7,8,9,10]. However, each block has a pre-determined translucency, which, added to the numerous shades available, necessitates quite a large stock.

One of lithium disilicate’s most appreciated clinical qualities is its aesthetics, a pivotal factor in restorative dentistry. Among the optical properties, translucency is essential for achieving a pleasing and tooth-like appearance [11,12,13], as well as minimizing the influence of a discolored substrate [14]. Therefore, translucency is a key feature of ceramic materials, including porcelain, lithium disilicate, and zirconia, and it plays a critical role in material selection [15,16].

The crystallization of CAD/CAM blocks to obtain lithium disilicate (Li_2_Si_2_O_5_) is a heat-mediated process that follows milling [17,18], and it is influenced by nucleating agents, which control crystal shape and size. Lithium disilicate crystals typically range from 1 to 3–4 μm and can exhibit round, plate-like, or rod-like morphologies, with manufacturers adjusting these parameters to optimize the trade-off between strength and translucency [2,19]. The crystallization process involves two major steps: nucleation and crystal growth [20,21]. The type and concentration of nucleating agents determine the final microstructure of the glass ceramic, affecting its mechanical and aesthetic properties [22,23]. Increasing the volume fraction of crystals typically improves strength and toughness [23] but negatively impacts translucency. Crystals act as an obstacle to the passage of light [24,25], promoting incoherent scattering due to the difference in the refractive index between the glass and crystalline phases, accentuated by the birefringence of the lithium disilicate crystals, which exhibit strong anisotropy in both their crystallographic structure and microstructure [26].

With the expiration of early patents, new lithium disilicate-based materials, such as Initial LiSi (GC), Cerec Tessera (Dentsply Sirona), and Amber Mill (HassBio), have entered the market. According to the manufacturer, Amber Mill uniquely offers customizable translucency through specific firing conditions, where temperature and holding time can be adjusted to influence the material’s final appearance [3,27]. However, while the manufacturer claims that these firing protocols allow for significant control over translucency without compromising flexural strength, few independent studies have validated these claims.

This study aims to critically assess whether the firing conditions produce differences in translucency without affecting the material’s flexural strength. The formulated null hypotheses are that varying the firing conditions to obtain different translucencies, according to the manufacturer’s indications, (i) does not significantly affect the material’s flexural strength and (ii) does not result in significant differences in the material’s translucency.

## 2. Materials and Methods

**Figure 1:**
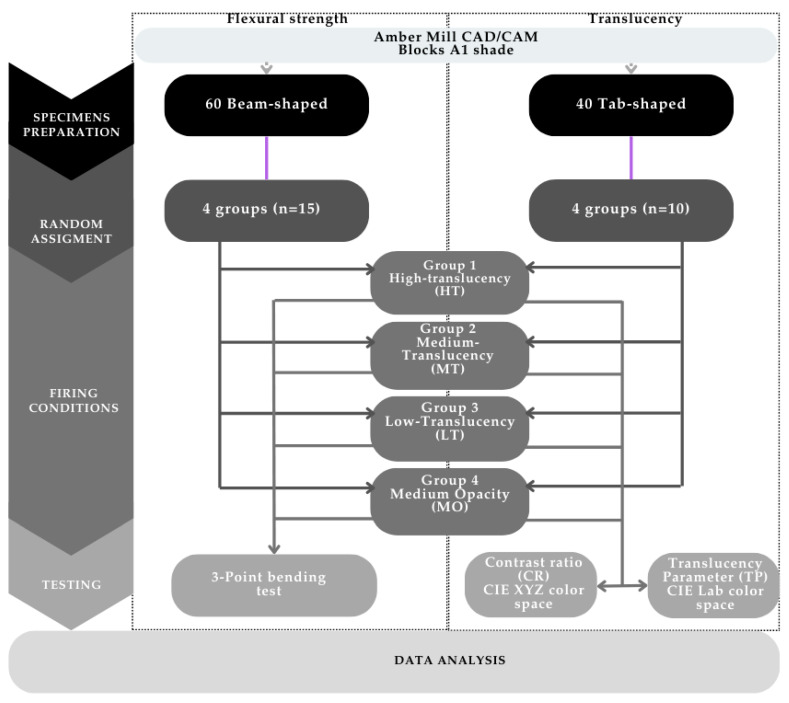
Experimental flowchart.

This study employed a quantitative research design to assess the effects of varying heating protocols on the flexural strength and translucency of one CAD/CAM material (Amber Mill, Haas Bio, Gangneung-si, Republic of Korea) (Figure 1). The blocks of the material were C14 size and A1 Vita shade. Using a proprietary device, the blocks were cut to obtain the desired shape depending on the test to be performed, flexural strength or translucency. The composition of the materials is reported in Table 1.

### 2.1. Flexural Strength

For the 3-Point Bending Test (3-PBT), sixty beam-shaped specimens were cut using a water-cooled cutting machine (low-speed saw, Buehler, Lake Bluff, IL, USA) and wet-finished in a grinder/polisher machine (Minimet, Buehler, Lake Bluff, IL, USA) with 600 grit paper until dimensions of 1.2 ± 0.2 mm in thickness, 4.0 ± 0.2 mm in width, and 15.0 ± 0.2 mm in length were obtained. Specimens were subsequently wet polished with 1200 and 2400 grit paper.

According to ISO 6872:2024, a 45° edge chamfer was made at each major edge [28]. After cutting, the Amber Mill specimens were randomly divided into four groups (n = 15) and submitted to four different crystallization firing processes in a ceramic furnace (VITA Vacumat 6000M, VITA Zahnfabrik, Bad Sackingen, Germany), following the Amber Mill manufacturer’s instructions to achieve the four different translucencies. The four crystallization processes (firing conditions) are reported in Table 2.

After firing, specimens were ultrasonically cleaned in distilled water for 10 minutes before the measurement procedure. Tests were performed using a universal testing machine (ESM 301, Mark-10, Copiague, NY, USA) equipped with a 50 N load cell (M5-50, Mark-10, Copiague, NY, USA) at a crosshead speed of 1 mm/min. The span was set at 13.0 mm. Specimens were tested dry at room temperature. The fracture load was recorded in N, and the flexural strength (σ) was calculated in MPa by using the following equation:

$$\sigma =\frac{3Pl}{2wb^{2}}$$

where *P* is the fracture load in N, *l* is the distance between the center of the supports in mm, *w* is the width in mm, and *b* is the height in mm.

The Weibull characteristic strength (σ_0_) and the Weibull modulus (m) were calculated according to the following equation:

$$P_{f}=1-exp\left[-{\left(\frac{\sigma }{\sigma_{0}}\right)}^{m}\right]$$

where *P_f_* is the probability of failure between 0 and 1, *σ* is the flexural strength in MPa, *σ*_0_ is the Weibull characteristic strength in MPa, and *m* is the Weibull modulus.

### 2.2. Translucency Measurement

For optical evaluation, forty tab-shaped specimens with final dimensions of 15.0 ± 0.5 mm in length, 15.0 ± 0.5 mm in width, and 1.0 ± 0.1 mm in thickness were obtained by cutting blocks perpendicularly to the long axis and wet polishing with 600 and 1200 grit paper. The specimens were then randomly divided into four groups (n = 10) and fired according to the four crystallization processes reported in Table 2. The specimens were then ultrasonically cleaned in distilled water for 10 min before the measurement procedure. The measurements were performed with a colorimeter (Color Meter Pro, Vetus Technology Co., Hefei, China) with an 8mm aperture and D/8 viewing geometry, which was calibrated for white and black following the manufacturer’s instructions. The colorimeter was connected to a smartphone running a dedicated app (ColorMeter V2.3.3). D65 illumination and a 10-degree standard observation angle were selected. Data were recorded in the CIEXYZ and CIELab colorimetric systems.

For every specimen, the Contrast Ratio (CR) was calculated with the following equation [14]:

$$CR=\frac{Yb}{Yw}$$

The Translucency Parameter (TP) was calculated using the following CIELab formula [30]:

$$TP=\sqrt[{2}]{{\left(L_{B}^{*}-L_{W}^{*}\right)}^{2}+{\left(a_{B}^{*}-a_{W}^{*}\right)}^{2}+{\left(b_{B}^{*}-b_{W}^{*}\right)}^{2}}$$

For both formulas, the “W” refers to values on a white background, while the “B” refers to values on a black background.

### 2.3. Scanning Electron Microscopy Observation

Two extra specimens per group were produced in the same way as the translucency specimens for microscopic evaluation of the glass ceramic microstructure. 

Specimens were etched for 120 s with 9% hydrofluoric acid (Porcelain Etch, Ultradent, South Jordan, UT, USA) and then cleaned under running water.

The preparation for SEM observations involved ultrasonic cleansing in a 95% alcohol solution for 3 min and air drying with an oil-free air spray.

Specimens were then secured onto SEM (Tescan MIRA 3, Brno, Czech Republic) slabs using gold conducting tape and gold coated in a vacuum sputter coater (Quorum Q150R sputter coater, Quorum Technologies, Laughton, UK).

The treated surfaces were then observed at 20,000× magnification to evaluate the crystal morphology.

### 2.4. Statistical Analysis

The normality of data distribution was assessed using the Kolmogorov–Smirnov test, and the homogeneity of variances was verified with Levene’s test. For flexural strength and the Translucency Parameter (TP), the data met the assumptions for parametric analysis; thus, a one-way ANOVA was performed, followed by the Tukey test for post hoc comparisons. In contrast, for Contrast Ratio (CR), the assumption of homogeneity of variance was not satisfied; therefore, a Kruskal–Wallis one-way ANOVA on ranks was applied, followed by the Tukey test for post hoc analysis. The level of significance was set at *p* < 0.05 for all statistical tests. Analyses were conducted using SigmaPlot 11.0 (Systat Software, Inc., San Jose, CA, USA).

## 3. Results

### 3.1. Flexural Strength

The flexural strength results are presented in Table 3. The ANOVA showed a statistically significant difference among the four tested groups (*p* < 0.001). MO showed the highest flexural strength values, although they were not statistically different from those of LT. HT showed the lowest results, but there was no statistically significant difference compared with MT. LT and MT did not show a statistically significant difference (Table 3).

The Weibull analysis of the flexural strength is presented in Table 3.

Amber Mill MO obtained the lowest Weibull modulus (m), and MT obtained the highest.

**Figure 2:**
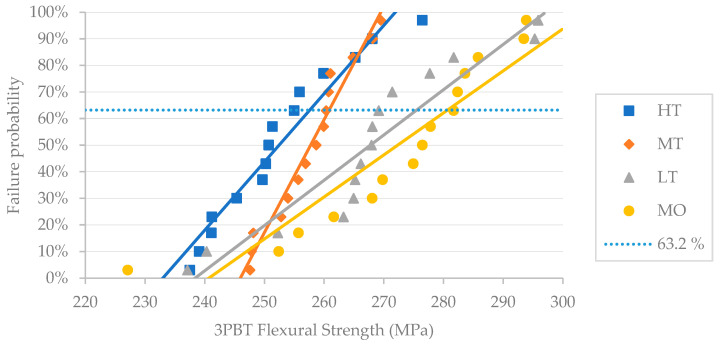
Failure probability of Amber Mill HT, MT, LT, and MO according to the Weibull analysis of 3PBT flexural strength. Failure probability at 63.2% corresponds to the Weibull characteristic strength (σ_0_). Fitting lines by method of least squares depict Weibull modulus (m).

Concerning Weibull characteristic strength, MO showed the highest *σ*_0_, while HT exhibited the lowest. Figure 2 displays the calculated data for the fracture results of 15 specimens in each group, along with the fitting of the dataset for each material using the least-squares method; the best-fit straight line with the smallest error determines the failure probability line for each material. According to the fitting line, MO can withstand the maximum force, while HT can withstand the lowest.

### 3.2. Translucency–Contrast Ratio

The four different crystallization firing processes resulted in statistically significant differences in translucency (*p* < 0.001). Increasing CR values were in agreement with the declared opacities of the specimens. However, statistically significant differences were found only between Amber MO and the other groups (Table 4).

### 3.3. Translucency—Translucency Parameter

The results obtained for TP are presented in Table 5. Decreasing TP values were observed, which were related to decreasing degrees of translucency. Similar to the values obtained by analyzing CR, significant differences between groups were observed only between Amber MO and the other groups (*p* < 0.001) (Table 5).

### 3.4. Scanning Electron Microscopy Observation

**Figure 3:**
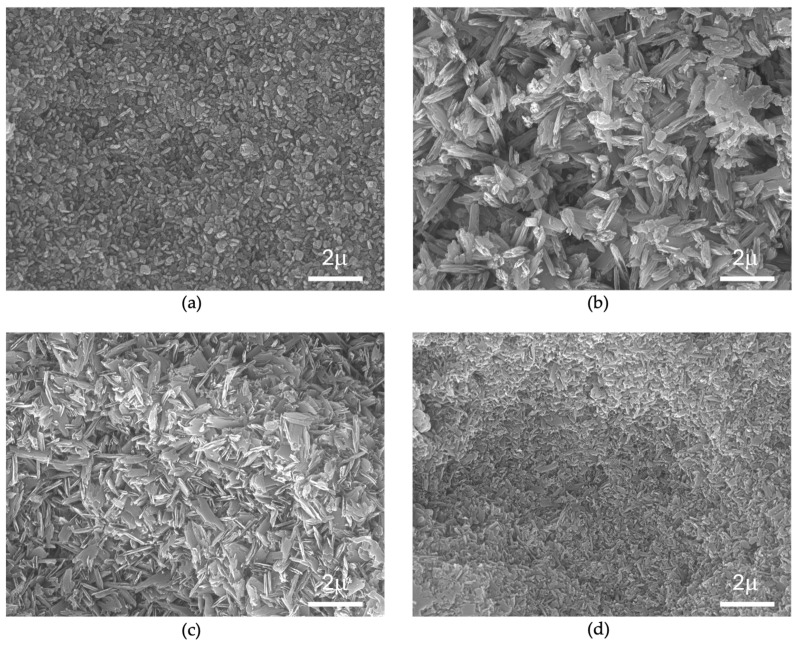
SEM images of Amber Mill after different heat treatment protocols. (20,000×). (**a**) Amber Mill HT; (**b**) Amber Mill MT; (**c**) Amber Mill LT; and (**d**) Amber Mill MO. Different crystal shapes and sizes are observed, with Amber MT and LT exhibiting larger and elongated particles compared to Amber HT and MO.

Scanning electron microscopy images of Amber Mill, subjected to four different firing processes, are presented in Figure 3.

The Amber Mill HT image displays small oblate- and plate-like crystals, which contrast with the larger and more densely packed crystals exhibited by Amber MT and Amber LT, where clearer boundaries between the crystals and matrix (removed by acid etching) are observed. Amber MO presented a denser and irregularly shaped crystal microstructure.

## 4. Discussion

In CAD/CAM procedures, materials like Amber Mill are provided in a partially crystallized state with moderate strength, allowing for easier milling [31,32,33,34,35]. After milling, heat treatment in a furnace converts the material into its final form, which consists of glass and crystalline phases. Different heat treatment protocols affect the growth of lithium disilicate crystals and the percentage of residual glass [35]. However, when oxides such as phosphorous pentoxide (P_2_O_5_), zirconium dioxide (ZrO_2_), vanadium pentoxide (V_2_O_5_), and cerium dioxide (CeO_2_) [8] are present in the composition, they act as co-nucleating agents to achieve the desired shade and translucency. These oxides interact with the nucleation and crystallization processes, altering crystal size and, thus, impacting the ceramic’s physical and mechanical properties [36]. When higher concentrations of nucleating agents are used, the microstructure becomes denser, and the crystals form smaller and rounder shapes [37], leading to reduced light scattering and increased opacity [11,38]. Although adding pigments can improve opacity without altering flexural strength [39], the final translucency depends more on crystal size and volume than specific compounds.

This study compared four different translucencies by applying four firing protocols to CAD/CAM blocks of a single shade of Amber Mill Nano-Lithium Disilicate. These protocols adhered to the manufacturer’s recommended heat treatment factors to achieve the desired translucency. The results suggest that the firing processes used to obtain lower translucency/higher opacity are linked to increased flexural strength. The statistical analysis led to the rejection of the first null hypothesis, as it revealed significant differences in flexural strength among the various translucencies.

Additionally, and differing from the manufacturer’s indications for the material, the recorded flexural strength values did not meet the ISO 6872:2024 clinical recommendations for ISO class 3 (three-unit bridge not involving molar restoration), as none of the groups surpassed the necessary threshold of 300 MPa [28]. However, it should be emphasized that the type of flexural strength test for class determination is not reported in the aforementioned ISO standards. Indeed, while test methods like flexural strength are crucial for comparing brittle materials, it has been demonstrated that changes in the test method alone can significantly affect flexural strength values [40]. In the case of biaxial flexural strength (BFT), where a disk is loaded at the center, it is believed that this reduces the probability of edge failure [41] since ceramic specimens are highly sensitive to edge or surface machining damage [42]. In this study, a three-point bending test (3-PBT) was preferred over BFT, because it does not require milling, the specimens can be obtained by cutting the blocks, and also because it is more widely used in research, therefore it allows for easier comparison with other studies/materials. It is well known that BFT yields higher results for dental ceramics than 3-PBT [43,44]. Therefore, the outcome indicating that the values measured in the present study using 3-PBT are lower than those found in the literature for BFT [45,46] was expected. No studies other than the present one have previously tested Amber Mill with 3-PBT.

One of the primary strengthening mechanisms in glass ceramics is the interlocking of crystals within the microstructure [47,48]. Crack propagation tends to occur at the crystal-glass interface, resulting in fractures [49,50]. This process is conditioned by metal oxides present in the ceramic [27]. Unfortunately, the complete composition of oxides within the ceramics’ microstructure, which may explain variations in physical properties like flexural strength, is not fully disclosed by manufacturers, limiting the potential for understanding the reasons for the measured differences. However, differences in flexural strength could be attributed to the crystal structures observed in the SEM images, where Amber Mill MO showed fewer voids and had lost its elongated crystal structure.

As chewing forces are unevenly distributed, a single strength value like flexural strength alone does not fully reflect a material’s mechanical performance. In this regard, the Weibull analysis is a valid method for evaluating the reliability and lifespan of engineering materials, particularly brittle materials like dental ceramics, which must withstand chewing forces. Weibull analysis accounts for variability by determining the probability of failure under different pressures, thereby providing deeper insights into material properties [51]. In the Weibull analysis, the Weibull characteristic strength (σ_0_) indicates the 63.21st percentile of the strength distribution, while the Weibull modulus (m) represents flaw distribution. A higher m value suggests better structural reliability [44,52]. Generally speaking, it is preferable to have a higher m, even with a slightly lower mean fracture strength, than a lower m associated with a higher mean fracture strength. Materials with higher Weibull moduli exhibit a more uniform distribution of defects, which describes the lifetime and frequency of failure of brittle materials. Materials with m > 20 demonstrate high structural integrity and reliability [53]. In the present study, Amber Mill HT and MT obtained m values higher than 20 (26.25 and 44.55, respectively), suggesting greater structural integrity and reliability than Amber Mill LT and MO, although the latter showed values close to the reported threshold (respectively 19.37 and 18.43). It is interesting to note that although Amber Mill fired with the MO program presented the highest flexural strength, at the same time, it showed the lowest Weibull modulus. Simba et al. [26], comparing the effects of temperature on the mechanical properties of IPS e.max CAD, found a similar reduction in the Weibull modulus at increasing temperatures due to the transient phases of cristobalite, lithium metasilicate, and lithium disilicate, which might have caused localized residual stresses, resulting in a wider spread of the flexural strength values.

According to the manufacturer, Amber Mill’s translucency can be controlled by firing at different temperatures. To achieve high translucency, the final crystallization temperature should be set at 815 °C; for medium translucency, it should be 825 °C; for low translucency, it should be 840 °C; and for medium opacity, it should be 860 °C. These variations in firing temperatures cause changes in crystal distribution and density, which affect visible light transmission. This ability to adjust translucency by choosing the firing temperature offers clinicians great flexibility and reduces stock requirements [10]. However, the results obtained in the present study differ from those presented by the manufacturer, with only Amber MO achieving significant differences compared to the other translucencies.

Thus, the second null hypothesis was rejected.

Translucency refers to the amount of light that passes through a material, where it is scattered, reflected, or transmitted [54]. Higher light transmission results in greater translucency. The translucency of dental ceramics increases as thickness decreases, showing a significant relationship between material type and thickness [55]. Two parameters are commonly used to measure the translucency of dental materials: the Translucency Parameter (TP) [13,30,56,57,58,59,60] and the Contrast Ratio (CR) [12,58,61,62]. TP is the difference between the reflected colors of a material with uniform thickness viewed over black and white backgrounds, providing a direct perception of translucency [30]. CR is the ratio of reflectance over black to that over white, representing opacity [63]. CR values range from 0 to 100, where 0 indicates total translucency, and 100 indicates total opacity [54]. 

Ideally, different thicknesses should be tested to better represent the material’s optical behavior. 

According to Liu et al. [64], CR differences below 7 are not perceptible to the human eye, based on the mean translucency perception threshold (TPT), although experienced clinicians may have a TPT of 4.

In this study, the CR differences for HT (52.6 ± 3.9), MT (54.3 ± 4.1), and LT (56.4 ± 2) were below 7, indicating no visible differences in translucency for a layperson. In contrast, HT and MT showed a CR difference greater than 7 when compared to MO (63 ± 1.8). LT showed a difference slightly lower than 7, but very close to this threshold. Comparing these values to those reported by Dietschi et al. [65], MO’s CR is similar to dentine (CR 62 to 70), while HT, MT, and LT fall between enamel (CR 40 to 49) and dentine.

No TPT has been identified for the Translucency Parameter yet. However, the study by Yu et al. [66] reported TP values for 1 mm-thick human enamel and dentin of 18.7 and 16.4, respectively. Therefore, according to those values, for the Translucency Parameter, only MO showed values approximately intermediate between the two natural tissues, while LT, MT, and HT showed higher translucency at the same thickness.

Understanding the reasons for translucency differences among materials is quite complex. SEM observation provides limited insight, as interactions between crystal size, shape, number, and the glass phase play a role in translucency [53]. Crystal size influences translucency, as light transmission through lithium disilicate ceramics is disrupted by crystals [67]. Phark and Duarte [8] reported that Amber Mill’s crystal size is 0.2 μm, which is quite small compared to that of other lithium disilicates; however, they did not report which firing protocol was used in their study. Jurado et al. [68] found differences in translucency between lithium disilicate crowns subjected to one and two sintering processes. In the same paper, while IPS e.max CAD translucency increased after multiple firings, Amber Mill’s translucency was reported to decrease, possibly due to its higher glass composition and smaller crystal size. 

These can be considered relevant, but they provide little information, and further studies are necessary to expand the knowledge on the optical behavior of glass ceramic materials.

## 5. Conclusions

- The use of different firing protocols resulted in different translucencies of the tested material (Amber Mill).
- The translucencies obtained are ranked according to the manufacturer’s indications. However, the differences between HT, MT, and LT were statistically not significant for both CR and TP and were below the available thresholds for translucency perceptibility. Only MO was significantly more opaque than the other three translucencies and above the threshold.
- The different translucencies obtained showed statistically significant differences in flexural strength when measured using the 3-PBT.

The manufacturer’s approach, which aimed to reduce the need for multiple stocks of translucencies through firing protocols, was only partially successful. Further studies exploring the influence of different firing protocols on other mechanical characteristics, like fracture toughness and fatigue, are advisable.

## Figures and Tables

**Table 1:** Characteristics of the material tested.

Material	Acronym	Manufacturer	Translucency *	Chemical Composition [8] ^±^	Definition	Thermal Treatment	Batch
Amber Mill	AM	Hassbio, Gangneung-si, Republic of Korea	HT, MT, LT, MO	<78% SiO_2_; <12% Li_2_O; <12% coloring oxides	Lithium disilicate	Yes	EBE05ND0801

* According to the manufacturer, different translucencies are achieved by selecting the corresponding translucency heat treatment. HT: high translucency, MT: medium translucency, LT: low translucency, MO: medium opacity. ^±^ Chemical composition stated by the manufacturer.

**Table 2:** Recommended crystallization heat treatment [29].

	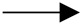	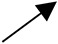		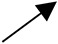	T		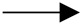	VAC		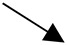
Predrying Temperature	Predrying Time	Heating Time		Temperature Rise Rate	End Temperature		Holding Time	Vacuum Holding Time		Long-Term Cooling
°C	min.	min.		°C/min.	°C		min.	min.		°C *
400	3.00	HT	6.50	60	HT	815	15.00	HT	21.50	690
		MT	7.05		MT	825		MT	22.05	
		LT	7.20		LT	840		LT	22.20	
		MO	7.40		MO	860		MO	22.40	

* The firing chamber must not be opened during long term cooling.

**Table 3:** Results of tested materials ordered by flexural strength.

		Flexural Strength		Weibull Statistics	
Material	Firing Protocol	*σ* (MPa)	*Sig*	*m*	*σ*_0_ (MPa)
Amber Mill	MO	272.31 ± 17.57	a	18.43	279.96
Amber Mill	LT	267.74 ± 16.53	ab	19.37	274.53
Amber Mill	MT	257.73 ± 6.91	bc	44.55	260.61
Amber Mill	HT	252.45 ± 11.29	c	26.24	256.89

*σ* = Flexural strength (mean and standard deviation); *Sig* = Significance; *m* = Weibull modulus; *σ*_0_ = Weibull characteristic strength. The same letter of significance indicates no statistically significant difference.

**Table 4:** Results of tested materials ranked by translucency (CR).

		Translucency	
Material	Firing Protocol	*CR*	*Sig*
Amber Mill	HT	52.6 ± 3.9	a
Amber Mill	MT	54.3 ± 4.1	a
Amber Mill	LT	56.4 ± 2.0	a
Amber Mill	MO	63.0 ± 1.8	b

*CR* = Contrast Ratio (mean ± standard deviation); *Sig* = Significance; same letter of significance indicates no statistically significant difference (*p* < 0.001).

**Table 5:** Results of tested materials ranked by translucency (TP).

		Translucency	
Material	Firing Protocol	*TP*	*Sig*
Amber Mill	HT	21.7 ± 1.9	a
Amber Mill	MT	21.2 ± 1.6	a
Amber Mill	LT	20.1 ± 1.1	a
Amber Mill	MO	17.5 ± 1.1	b

*TP* = Translucency Parameter (mean ± standard deviation); *Sig* = Significance; same letter of significance indicates no statistically significant difference (*p* < 0.001).

## Data Availability

The data presented in this study are available upon request from the corresponding author. Due to the university’s policy on access, they are not publicly available.

## Abbreviations

The following abbreviations are used in this manuscript:

TP	Translucency Parameter
CR	Contrast Ratio
3-PBT	Three-Point Bending Test
HT	High Translucency
MT	Medium Translucency
LT	Low Translucency
MO	Medium Opacity
CAD/CAM	Computer-Aided Design/Computer-Aided Manufacturing
SEM	Scanning Electron Microscopy
TPT	Translucency Perception Threshold
